# Self-Assembly TiO_2_-Ti_3_C_2_T_x_ Ball–Plate Structure for Highly Efficient Electromagnetic Interference Shielding

**DOI:** 10.3390/ma17010072

**Published:** 2023-12-22

**Authors:** Zhen Zhang, Xingyang Ning, Bin Liu, Jian Zhou, Zhimei Sun

**Affiliations:** 1 School of Materials Science and Engineering, Beihang University, Beijing 100191, China; by1901053@buaa.edu.cn (Z.Z.); 13032495593@163.com (X.N.); jzhou@buaa.edu.cn (J.Z.); 2 School of Integrated Circuit Science and Engineering, Beihang University, Beijing 100191, China; binliu@buaa.edu.cn

**Keywords:** electromagnetic interference shielding, MXene, TiO_2_-Ti_3_C_2_T_x_, ball–plate structure

## Abstract

MXene is a promising candidate for the next generation of lightweight electromagnetic interference (EMI) materials owing to its low density, excellent conductivity, hydrophilic properties, and adjustable component structure. However, MXene lacks interlayer support and tends to agglomerate, leading to a shorter service life and limiting its development in thin-layer electromagnetic shielding material. In this study, we designed self-assembled TiO_2_-Ti_3_C_2_T_x_ materials with a ball–plate structure to mitigate agglomeration and obtain a thin-layer and multiple absorption porous materials for high-efficiency EMI shielding. The TiO_2_-Ti_3_C_2_T_x_ composite with a thickness of 50 μm achieved a shielding efficiency of 72 dB. It was demonstrated that the ball–plate structure generates additional interlayer cavities and internal interface, increasing the propagation path for an electromagnetic wave, which, in turn, raises the capacity of materials to absorb and dissipate the wave. These effects improve the overall EMI shielding performance of MXene and pave the way for the development of the next-generation EMI shielding system.

## 1. Introduction

The electromagnetic wave produced using electronic equipment damages the device system, limits equipment performance, and even endangers human health. Therefore, electromagnetic interference (EMI) shielding materials, which can block electromagnetic waves within a specific frequency range based on reflection and internal dissipation absorption [1,2,3], are required to solve these threats. However, conventional shielding materials, such as metallic foil, cannot satisfy the requirements for a light weight and corrosion resistance. Developing novel and effective EMI shielding materials has, thus, become a challenge for researchers [4,5,6,7].

Over the years, researchers have discovered a series of new two-dimensional transition metal carbides and nitrides, known as MXene [8,9], which show excellent potential in lightweight EMI shielding performance owing to their low density [10], unique 2D nanosheet structures [11], high electrical conductivities [12,13], and film-forming performances (which are conducive to forming a continuous conductive network) [14,15]. In the meantime, a large number of polar groups (-O or -OH, etc.) are suspended on its surface, providing an abundance of active sites for the attachment of water molecules, nanoparticles, or magnetic units, rendering MXene with hydrophilic properties and the ability to modify polarization loss [16,17]. It is anticipated that MXene will become a broadly applicable EMI shielding material. 

Experiments have revealed that multilayer-stacked MXene flakes have an admirable EMI shielding ability due to the impedance mismatch between the high-conductivity substrate and the low-conductivity air dielectric; nevertheless, their electromagnetic wave absorption capacities are relatively deficient [18]. Simultaneously, MXene nanosheets lack interlayer support and are prone to agglomeration, which will destroy the unique structure of MXene, reduce the electromagnetic wave absorption efficiency, and shorten the service period [6]. Therefore, researchers try to avoid the agglomeration of MXene by building superstructures, such as CNT/MXene aerogel [19], MXene/graphene [20], MXene/polymer inclusions [21], MXene/Ni Chain [22], RGO/Ga@PEDOT:PSS [23], etc. The superstructure can indeed substantially improve the EMI performance of MXene, albeit usually with an increase in sample thickness. Due to the specificity of the structure and filler, reducing the material thickness will significantly weaken shielding performance [4]. This structure–performance paradox limits the application of superstructures in the field of EMI shielding.

In MXene, Ti_3_C_2_T_x_ possesses high electrical conductivity, stable performance, and a brief manufacturing process [23,24,25,26,27], and it has been regarded as the preferred choice for EMI shielding material substrates [28,29]. Herein, we proposed and demonstrated a new multilayer porous ball–plate structure by using homogeneous and lightweight titanium dioxide (TiO_2_) hollow spheres as the support phase between samples of few-layer Ti_3_C_2_T_x_. The composite ball–plate structure obtained through a simple solution self-assembly method exhibits superior performance compared to previous studies under similar conditions (matrix and doped phase). This enhanced performance can be attributed to the gains derived from its unique structure. Additionally, the sample film obtained through filtration is only a few tens of micrometers thick, offering higher practical value compared to the millimeter-level thickness of most porous shielding materials [5,22]. This superstructure simultaneously complies with the demands of low thickness, less agglomeration collapse, and superior shielding performance, thereby establishing a new development direction for novel lightweight thin-layer EMI shielding materials.

## 2. Design Principles and Synthesis of the Ball–Plate EMI Shielding Materials

Our design principle is to discover composite materials that possess both low density and high electrical conductivity while optimizing their electromagnetic shielding effectiveness through structural design. Firstly, for EMI shielding materials, the electrical conductivity, dielectric loss, and magnetic permeability all play significant roles in adjusting impedance matching and improving electromagnetic wave attenuation [30,31]. It was found that the dielectric loss effect is contributed by polarization loss and conductance loss [32], whereas the magnetic loss is primarily contributed by the magnetic component [22]. Therefore, to regulate the dielectric properties of a composite material, the selection of a filler with a high dielectric loss is crucial. Secondly, to preserve the heterogeneous interface and maintain a stable interlayer structure, it is essential to ensure that the filler forms a strong connection with the substrate (via van der Waals forces, covalent bonding, etc.). Thirdly, to improve the efficiency of electromagnetic wave dissipation, the design of porous foam and hollow structure inside the material is a reliable choice [33,34]. Fourthly, to increase the polarization interface and extend the electromagnetic wave transmission path, void structures should be constructed within the composite material. This can be achieved by selecting an optimal combination of filler materials and particle sizes, which create channels for wave propagation.

**Figure 1:**
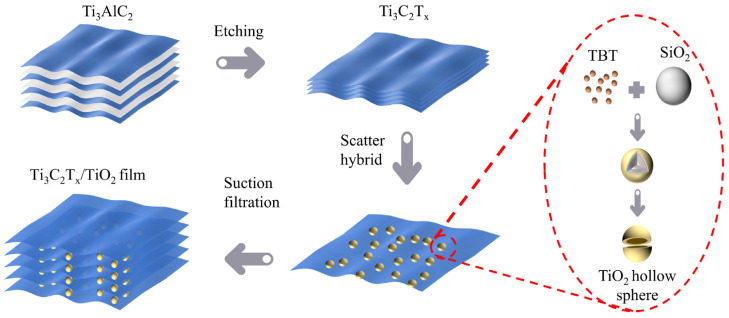
Schematic diagram of the fabrication method for the TiO_2_-Ti_3_C_2_T_x_ composite.

After evaluating the candidate materials and structures based on the prescribed criteria, we have identified a promising option for EMI shielding: the multilayer ball–plate structure composed of TiO_2_ and Ti_3_C_2_T_x_. This structure exhibits excellent properties owing to the high dielectric loss effect of TiO_2_, which can enhance microwave absorption performance. Additionally, the surface of Ti_3_C_2_T_x_ contains dangling bonds that can easily connect with TiO_2_, enabling the self-assembly and stable combination of the composite material. To satisfy the requirements for application and testing, vacuum filtration is employed to obtain the thin-layered shielding materials that have been utilized in the majority of studies [34]. We, firstly, etched MAX Ti_3_AlC_2_ by using HCl and LiF to obtain the desired monolayer MXene material, i.e., Ti_3_C_2_T_x_. Subsequently, hollow titanium dioxide spheres were prepared via the template method, and SiO_2_ is used as the liner. Finally, the above materials were combined thoroughly to obtain a suspension. After vacuum filtration and freeze-drying, a material with a flexible lamellar structure was produced. Preparation details can be found in Appendix A. The overall preparation process is shown in Figure 1.

## 3. Results and Discussion

### 3.1. Structure Characterization

**Figure 2:**
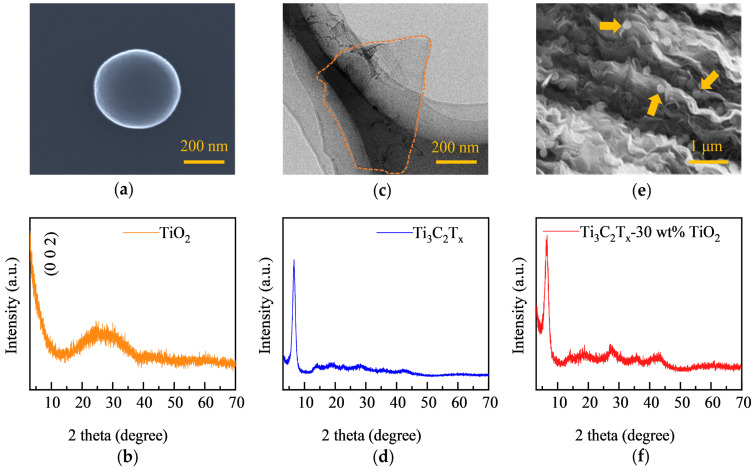
(**a**,**b**) SEM and XRD pattern of the monodisperse amorphous TiO_2_ hollow spheres. (**c**,**d**) TEM image and XRD pattern of the Ti_3_C_2_T_x_ MXene flakes (marked by dotted lines). (**e**,**f**) Cross-sectional SEM images and XRD pattern of the TiO_2_-Ti_3_C_2_T_x−_30 wt.% composites. The TiO_2_ hollow spheres in the layer are marked by arrows.

To construct the target superstructures, we synthesized TiO_2_ hollow spheres and Ti_3_C_2_T_x_ MXene and characterized their microstructures. The scanning electron microscope (SEM) morphology of the TiO_2_ ball is shown in Figure 2a. The TiO_2_ hollow spheres, prepared with a diameter of 180–220 nm and a thickness of approximately 20 nm, exhibit uniform and monodisperse characteristics after stirring and ultrasonication, aligning with the results outlined in the referenced literature [35]. Figure 2b depicts the X-ray diffraction (XRD) pattern of the prepared TiO_2_, which exhibits a characteristic amorphous steamed bread peak between 15° and 40°. The homogenous, hollow, and amorphous properties of the monodispersed TiO_2_ ball are further investigated using the transmission electron microscope (TEM, Figure S1). The microstructure of the monolayer Ti_3_C_2_T_x_ MXene is given in Figure 2c. An atomic force microscope (AFM) analysis was conducted to establish the lamellar thickness of Ti_3_C_2_T_x_. It is shown that the thickness of the Ti_3_C_2_T_x_ is 2.2 nm, which is consistent with the calculated results [29], suggesting that it is a single layer (Figure S2). Compared to the original MAX phase Ti_3_AlC_2_, the characteristic peak of the Al atomic layer (at 39.1°) was eliminated from the Ti_3_C_2_T_x_ XRD spectrum (Figure 2d), and the position of the main peak was moved from 9.5° to 6.54°, indicating that the desired MXene was properly synthesized. Figure 2e,f display the cross-sectional morphology and XRD patterns of TiO_2_-Ti_3_C_2_T_x_ composites with 30 wt.% TiO_2_ (termed as TiO_2_-Ti_3_C_2_T_x−_30 wt.%). It is clear that the TiO_2_ hollow spheres are evenly distributed among the layers of Ti_3_C_2_T_x_ and spontaneously adsorb on the surface of the Ti_3_C_2_T_x_ film, preventing excessive stacking of Ti_3_C_2_T_x_ flakes. Due to TiO_2_ support, spaces that form between Ti_3_C_2_T_x_ layers are conducive to improving the electromagnetic wave in its internal losses [36]. The characteristic peaks of TiO_2_ and Ti_3_C_2_T_x_ were both observed in the XRD pattern, which means that TiO_2_ and Ti_3_C_2_T_x_ have achieved a structural combination. The characterization of other TiO_2_-Ti_3_C_2_T_x_ composites is available in Figures S5 and S6. With the increase in TiO_2_ content, TiO_2_ hollow spheres appear to aggregate. Moreover, the prepared TiO_2_-Ti_3_C_2_T_x_ material has a thickness of only 50 μm, and the assessed TiO_2_-Ti_3_C_2_T_x_ films demonstrated remarkable flexibility, exhibiting no discernible signs of damage after cyclic bending of 180° for 3000 iterations. Simultaneously, the tensile strength experiences a marginal reduction upon the incorporation of TiO_2_, remaining as 70% of the pure Ti_3_C_2_T_x_. This slight reduction in tensile strength is of relatively minimal consequence for the compound’s intended use as a thin-layer coating material (Figure S3).

**Figure 3:**
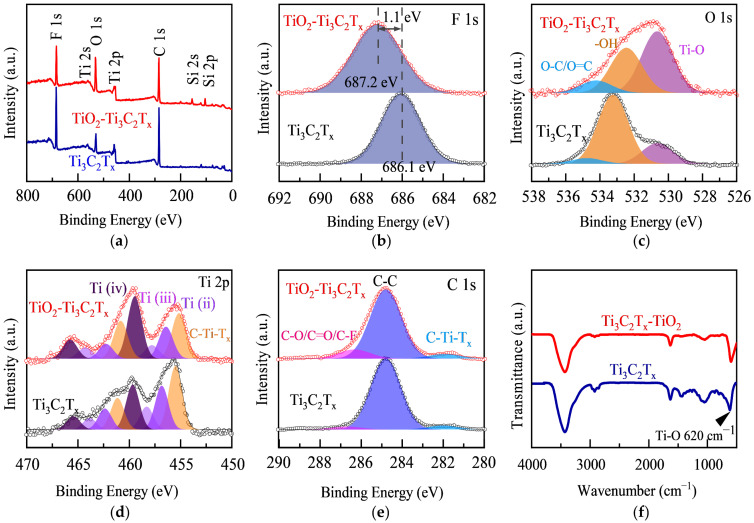
XPS patterns of Ti_3_C_2_T_x_ MXene and TiO_2_-Ti_3_C_2_T_x−_30 wt.% composites, (**a**) full spectrum, (**b**) F 1s, (**c**) O 1s, (**d**) Ti 2p, and (**e**) C 1s. (**f**) FTIR patterns of Ti_3_C_2_T_x_ MXene and TiO_2_-Ti_3_C_2_T_x−_30 wt.% composites.

X-ray photoelectron spectroscopy (XPS) was employed to investigate the chemical composition and bonding environment of TiO_2_-Ti_3_C_2_T_x−_30 wt.% composites. Compared to the full XPS spectrum of original Ti_3_C_2_T_x_ MXene, the composite material has a significant Si signal (Figure 3a) originating from the mold of TiO_2_ hollow spheres, which further confirms the existence of the hollow sphere TiO_2_. Furthermore, the incorporation of oxides into the composite leads to a marked increase in the O 1s peak intensity, as well as a relative decrease in the F 1s peak intensity compared to the original Ti_3_C_2_T_x_ MXene (Figure 3b,c). The F element in the composite exhibits a shift toward higher binding energy (Figure 3b), indicating the formation of the chemical bond between TiO_2_ and Ti_3_C_2_T_x_ via the replacement of some original Ti-F bonds by Ti-O bonds. The remaining fluorine-containing groups cause a strong dipole polarization effect, which is beneficial for the attenuation and absorption of the electromagnetic wave energy of the composites. As expected, the XPS pattern of the C element exhibited relatively slight variation across multiple samples due to its lesser involvement in surface bonding. In addition, the FTIR (Fourier transform infrared reflection) peak shapes of the Ti_3_C_2_T_x_ MXene and TiO_2_-Ti_3_C_2_T_x−_30 wt.% composites are nearly identical, with no discernible differences in peak positions (Figure 3f). However, the difference in the peak shape of the corresponding Ti-O transmission peak at 620 cm^−1^ indicates that the chemical environment of Ti-O bonding has changed.

The relative contents of each element in different functional groups/covalent bonds were further analyzed to elucidate the interface characteristics of the filler and matrix in the composite material (Table 1). The Ti-O bond content in the Ti element of Ti_3_C_2_T_x_ is 23.0%, while the Ti-O content in the TiO_2_-Ti_3_C_2_T_x−_30 wt.% material is 34.5%, indicating that Ti-O bonds contribute to 51.7% of the oxygen in the composite, which is 2.4 times higher than that of Ti_3_C_2_T_x_. These findings demonstrated that the composites contain a significant degree of chemical bonding between TiO_2_ and Ti_3_C_2_T_x_ rather than simple mechanical mixing. In other words, the O element in TiO_2_ forms a new bond with the Ti element in Ti_3_C_2_T_x_, and the matrices of the composite material and the filler are successfully recombined at the nanometer scale. Moreover, the composites exhibit a distinctive architecture, consisting of interleaved layers of few-layered Ti_3_C_2_T_x_ sheets and hollow spheres, which is in line with the morphological and structural characterizations determined in previous experiments (Figure 2).

### 3.2. EMI Shielding Performance

**Figure 4:**
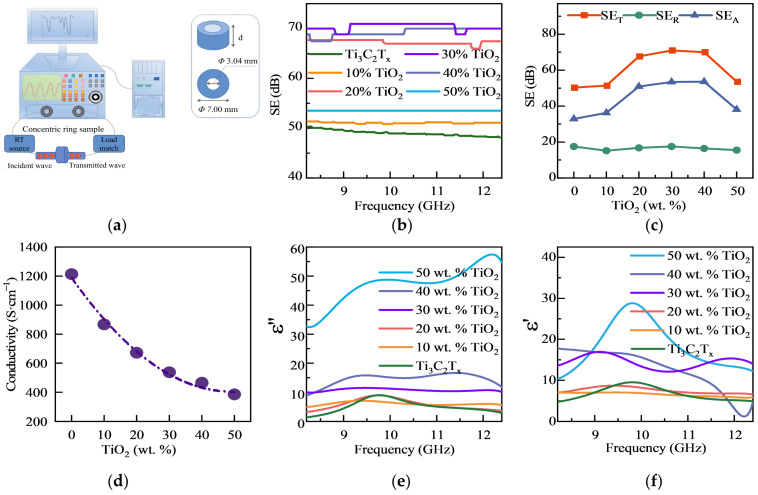
EMI shielding test methods and related data. (**a**) Schematic diagram of vector network analyzer sample and testing process. (**b**) SE_T_ of TiO_2_-Ti_3_C_2_T_x_ composites as a function of frequency. (**c**) SE_T_, SE_A_, and SE_R_ of TiO_2_-Ti_3_C_2_T_x_ composites under 12 GHz. (**d**) Conductivity of composites with different TiO_2_ contents. Imaginary (**e**) and real parts (**f**) of dielectric constants of composites with different TiO_2_ contents.

Generally, the shielding mechanism of layered materials is primarily attributed to the interaction of the incident EMI wave with the surface and interior of the film. When the electromagnetic wave interacts with the shielding layer, the impedance mismatch between the measuring material itself and the air results in part of the incident wave being reflected by the interface, while the remaining wave is absorbed by the shielding material and internally dissipated. According to the Schelkunoff theory, electromagnetic shielding absorption comprises three components, namely reflection, absorption, and multiple reflections [37,38,39]. The latter component is closely related to the thickness of the shielding material [40]. When the thickness of the material is much greater than the skin depth (~1 μm) or the electromagnetic shielding efficiency is greater than 15 dB, multiple reflections can typically be neglected [41]. Hence, the focus of our investigation is primarily on the absorption, reflection, and overall shielding effectiveness of the material. Based on the constructed test system and the displayed sample geometry (Figure 4a), the X-band frequency range (8.2 to 12.4 GHz) was utilized to measure the shielding properties of the composites. The frequency dependence of the SE_T_ (total shielding effectiveness) within each material was examined, as illustrated in Figure 4b, in the specified frequency range, and each material exhibits slight fluctuations in its SE_T_. However, there is a discernible trend in the average SE_T_ values of the materials in the X-band. Specifically, as the TiO_2_ content increases, the SE_T_ initially increases and then decreases. The maximum SE_T_ value 72 db is observed at a TiO_2_ content of 30 wt.%. To provide a better understanding of the shielding effect, the electromagnetic frequency of the wave was fixed. Figure 4c displays the SE_T_, SE_A_ (absorption shielding effect), and SE_R_ (reflection shielding effect) of every sample at a fixed frequency of 12 GHz. The SE_T_ and SE_A_ exhibit a similar trend of initially increasing and then decreasing with the increase in TiO_2_ content. In contrast, the SE_R_ shows relatively minor changes. To further investigate the influence mechanism, we separately examined the effects of absorption and reflection.

Firstly, reflection occurs at the interface between two electromagnetic wave propagation media with different impedance or refractive indices, which is one of the most significant EMI shielding mechanisms. The following equation can be used to describe this mechanism [42,43]:

(1) $$SE_{R}=20\log\frac{Z_{0}}{4Z_{\mathrm{in}}}=39.5+10\log\frac{\sigma }{2f\pi \mu }\propto \frac{\sigma }{\mu }$$

where *Z*_0_ is the free space impedance, *Z_in_* is the interface impedance, *σ* is the total conductivity, *f* is the frequency, and *μ* is the magnetic permeability. As discussed above, the electrical conductivity of non-magnetic thin-layer EMI shielding materials is strongly correlated with their reflective properties. Therefore, we investigated the electrical conductivity and SE_R_ of composite materials with various TiO_2_ contents (Figure 4c,d). The original Ti_3_C_2_T_x_ conductivity can exceed 1000 S/cm, providing a solid foundation for the potential reflection of the electromagnetic wave. With the increased content of TiO_2_, the hollow spheres gradually agglomerate among the layers (as shown in Figure S6), leading to a gradual decrease in conductivity (Figure 4c). Interestingly, the expected subsequent decrease of the SE_R_ of the composites was not observed (primarily unchanged). It can be attributed to the fact that the surface of the composites is mainly composed of Ti_3_C_2_T_x_ flakes, which are less influenced by the filler content (Figure 4b).

Secondly, the electromagnetic wave can be attenuated when they encounter a shielding material. This attenuation rate, denoted by *α*, is determined by the intrinsic properties of the shielding material. A higher *α* can be achieved by using materials with larger dielectric constants, permeabilities, and electrical conductivities [44]. This mechanism can be expressed using the following formula [45,46]:

(2) $$E=E_{0}e^{-\alpha }d$$

(3) $$\alpha =\omega \sqrt{\frac{\mu \varepsilon }{2}\left[\sqrt{1+{\left(\frac{\sigma }{\omega \varepsilon }\right)}^{2}}-1\right]}$$

where E_0_ is the initial electromagnetic wave energy, E is the electromagnetic wave energy absorbed by the shielding material, *ε* is the dielectric constant, *d* is the thickness of the plate, *σ* is conductivity, and *μ* is the magnetic permeability. The ability of a material to absorb an electromagnetic wave is related to its dielectric constant, as shown in Formulas (2) and (3). Under an alternating electric field, the dielectric constant comprises two components: the real and imaginary parts. The real part represents the ability to store electromagnetic energy, and the imaginary part represents the ability to dissipate the electromagnetic energy of materials, respectively. Experimental measurements show that the dielectric constant and loss factor increase with TiO_2_ content in TiO_2_-Ti_3_C_2_T_x_ materials. The loss factor exhibits a relatively low frequency dependence and increases monotonically in the X-band. The difference is negligible when the TiO_2_ content is less than 20 wt.%, but it becomes significant at 30 wt.% and 40 wt.% and reaches a maximum at 50 wt.% (Figure 4e). In contrast, the dielectric constant varies more irregularly with frequency, showing three distinct numerical steps at 0–20 wt.%, 30–40 wt.%, and 50 wt.% (Figure 4f). The observed phenomena can be attributed to the increase in the dipole polarization (Ti_3_C_2_T_x_ MXene is commonly over-etched during preparation, resulting in Ti vacancies [47]) and non-homogeneous interfaces between Ti_3_C_2_T_x_ and TiO_2_ [48], which result from the addition of TiO_2_.

However, the agglomeration of TiO_2_ hollow spheres as fillers can lead to substrate discontinuity and increased material defects, resulting in decreased EMI shielding performance. The loss factor of the composites increases with an increase in TiO_2_ content, but as the electrical conductivity decreases, these two factors, which have opposite effects on SE_T_, eventually lead to an optimal value. The TiO_2_-Ti_3_C_2_T_x−_30 wt.% material exhibits the highest SE_A_ value and a simultaneous maximum SE_T_ of 72 dB. However, in comparison to previous studies using high-conductivity matrices and fillers with high-energy-dissipation properties, such as SiO_2_@ Ti_3_C_2_T_x_, Ni@ Ti_3_C_2_T_x_, and polystyrene@ Ti_3_C_2_T_x_, which achieved a shielding efficiency of approximately 60 dB at a thickness of 1 mm or more, our study demonstrates a significant advantage in both thickness (50 µm) and shielding efficiency (72 dB). As shown in the Figure S7 and Table S2, TiO_2_-Ti_3_C_2_T_x_ exhibits remarkable competitiveness in both thickness and shielding efficiency dimensions. Since all studies utilized the self-assembled construction of raw materials with high conductivity and exhibited a heterogeneous interface connection, the observed performance differences cannot be attributed to the intrinsic properties of the materials. Therefore, the internal structure of the materials is likely the key factor affecting the observed performance differences [47,49,50]. Besides intrinsic properties such as conductivity and dielectric constant, multiple scattering effects within the composite material significantly contribute to the SE_T_. Materials with internal cavities can act as effective electromagnetic wave absorbers due to their complex microstructures, which provide multiple interfaces for wave reflection and scattering. The internal cavities act as resonant cavities that cause multiple scattering, increasing the path length of the wave through the material, which enhances the interaction between the electromagnetic wave and the polarized interfaces of the cavities, leading to an increase in the absorption loss efficiency [13,51]. Consequently, multiple scattering within the material can increase the absorption loss of the electromagnetic wave. The dissimilarities in multiple scattering effects suggest differences in the internal structure of the material.

**Figure 5:**
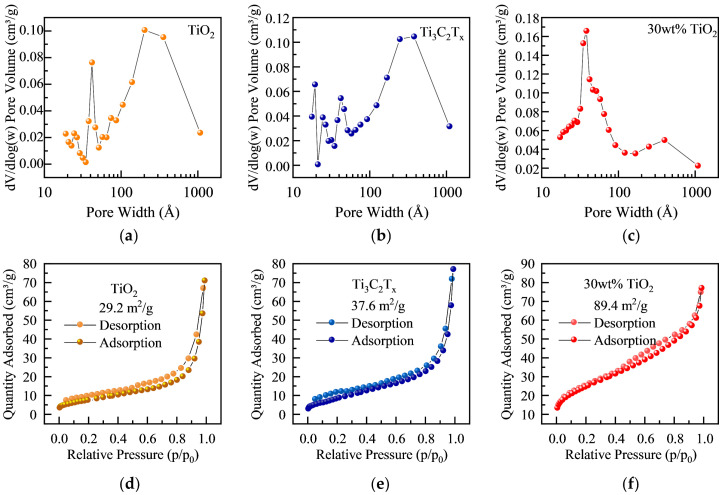
Pore size distribution diagrams of (**a**) TiO_2_, (**b**) Ti_3_C_2_T_x_, and (**c**) TiO_2_-Ti_3_C_2_T_x−_30 wt.%. Nitrogen adsorption–desorption isotherms of (**d**) TiO_2_, (**e**) Ti_3_C_2_T_x_, and (**f**) TiO_2_-Ti_3_C_2_T_x−_30 wt.%.

To further understand the internal structure of TiO_2_-Ti_3_C_2_T_x_ composites, nitrogen adsorption–desorption isotherm curves for samples with different TiO_2_ contents were studied to analyze the specific surface area and pore structure (Figure 5). It is shown that the added TiO_2_ significantly increased the specific surface area of the original Ti_3_C_2_T_x_ MXene. Furthermore, the TiO_2_ hollow spheres would spontaneously pin on the surface of the Ti_3_C_2_T_x_ film and prevent excessive stacking of Ti_3_C_2_T_x_ (Figure 5a–c). In addition, all nitrogen adsorption–desorption isotherms did not display a saturated adsorption platform, indicating an irregular pore structure. It can be attributed to the spherical pores provided by the hollow sphere filler, and numerous additional pores were generated between the TiO_2_ hollow spheres and their combinations with the Ti_3_C_2_T_x_ structure (Figure 5d–f).

The differential pore size distributions of three materials, namely TiO_2_ filler, Ti_3_C_2_T_x_, and their composite, were analyzed using the BJH (Barret–Joyner–Halenda) method. The TiO_2_ filler exhibits mesoporous and macroporous structures due to the hollow sphere structure and accumulation of spheres. Ti_3_C_2_T_x_ displays a hierarchical pore structure with micropores, mesopores, and macropores generated via in situ HF etching and the interlacing of multiple layers. The composite material has a similar hierarchical pore structure with increased mesopores and reduced macropores, indicating a uniform and proper combination of the basic materials. The decreased number of macropores was due to the filling of larger pores when Ti_3_C_2_T_x_ and TiO_2_ were separately stacked, and the distribution of the voids verified the proper combination of the materials in the microstructure. The composite material showed a significant increase in the number of mesopores, which exceeded the sum of mesopores in the matrix and filler. This indicates that combining Ti_3_C_2_T_x_ and TiO_2_ led to the creation of new mesopore-sized cavities in the material. In fact, the increase in porosity has two main effects. Firstly, the increase in pores is inevitably accompanied by an increase in internal interfaces within the material. At these interfaces, electromagnetic waves are further dissipated due to impedance mismatch and interface polarization losses, as reflected in the macroscopic increase in dielectric loss (Figure 4e,f). Secondly, the increase in porosity disrupts the continuity between MXene layers, consequently reducing the overall electrical conductivity of the composite material (Figure 4d). Ultimately, the enhancement in the total electromagnetic shielding performance of the composite material is attributed to the absorption gain resulting from the increased dielectric loss outweighing the reduction in reflection due to the decrease in conductivity. The combined effect of these pores resulted in the dissipation of electromagnetic wave inside the material, ultimately enhancing the electromagnetic shielding performance. The proposed ball–plate stack structure is an improvement over traditional multilayer plate and core–shell structures by providing additional cavities for multiple scattering effects, thereby enhancing the EMI shielding effectiveness.

**Figure 6:**
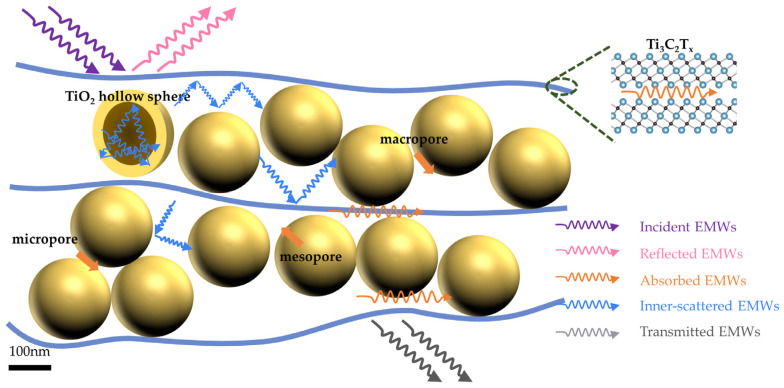
Schematic diagram of the interaction between the electromagnetic wave and interfaces, as well as the EMI shielding mechanism in TiO_2_-Ti_3_C_2_T_x_ composites.

Finally, we constructed a model to describe the electromagnetic shielding mechanism of the TiO_2_-Ti_3_C_2_T_x_ materials (Figure 6). Electromagnetic waves interact with materials through reflection, absorption, and transmission. The highly conductive MXene surface causes incident wave to reflect due to the impedance discontinuity at the air–material intersection. As the electromagnetic wave propagates within the material, it interacts with the TiO_2_ hollow sphere and TiO_2_-Ti_3_C_2_T_x_ heterogeneous interface, causing continuous attenuation and absorption. Meanwhile, the anchoring of MXene and TiO_2_ introduces new polarized Ti-O bonds, augmenting the dielectric loss performance of the material. These polarized bonds respond to an external electric field, inducing additional energy dissipation through the polarization and depolarization processes.

The ball–plate structure of the material creates numerous cavities, which increase the electromagnetic wave propagation path and form many impedances’ discontinuous interfaces. Upon encountering these interfaces, the remaining electromagnetic wave scatters multiple times and is ultimately almost entirely absorbed as eddy currents inside the MXene material, with only some of the wave being transmitted. This demonstrates that the excellent shielding efficiency is attributed to the superior electrical conductivity of MXene, the polarization interface inside the material, and the stable ball–plate structure. The high conductivity of Ti_3_C_2_T_x_ provides a high reflection efficiency for the electromagnetic wave, promoting the multiple scattering of interlayer pores. The abundant polar bonds between TiO_2_ hollow spheres and functional groups on the Ti_3_C_2_T_x_ surface facilitate electromagnetic wave absorption and dissipation. The ball–plate structure combines the benefits of porous foam and multilayer flat-plate structures to establish numerous pores that offer more reflection paths and polarization interfaces for the scattering and absorption of an electromagnetic wave. By incorporating appropriate fillers to support Ti_3_C_2_T_x_ flakes, a thin, flexible material with enhanced EMI shielding performance is obtained, eliminating the flaw that makes the interior area of the materials susceptible to collapse. The study indicates that to enhance the SE_T_ of compound materials, it is essential to consider the filler content and the filler morphology, size, and pore structure. Constructing non-homogeneous interfaces and mesoporous structures that match the fillers is crucial for achieving superior electromagnetic shielding performance. However, taking precautions is essential to avoid filler agglomeration and substantial conductivity drops.

## 4. Conclusions

In this study, we successfully synthesized a novel TiO_2_-Ti_3_C_2_T_x_ MXene composite material with a ball–plate structure via the self-assembly method. By adjusting the amount of TiO_2_ hollow spheres, it can tune the dielectric constant and EMI shielding effectiveness of the composite material. A sample with a thickness of only 50 μm containing 30 wt.% TiO_2_ exhibits a remarkable SE_T_ value of 72 dB. The excellent EMI shielding performance of the TiO_2_-Ti_3_C_2_T_x_ composite material can be attributed to its unique ball–plate structure, which provides multiple scattering of the electromagnetic wave due to the high electrical conductivity of the material and the interface polarization. The formation of pores with different sizes in the spherical–planar structure further increases the internal dielectric losses of the material, thereby enhancing the electromagnetic shielding performance. The combination of spherical filler and layered conductive matrix achieves a synergistic effect greater than the sum of its parts. And the unique structural factor will provide fresh insights for a range of studies on particle (metal oxide, metal clusters, carbon material particles) @ layered (MXene, MBene, metal foil, organic film) shielding composite materials.

## Figures and Tables

**Table 1:** The relative content of the corresponding functional groups/valent bonds of each element.

Element	Sample	Binding Energy (eV)	Binding	Relative Content
Ti 2p	Ti_3_C_2_T_x_	455.5 (461.2)	C-Ti-T_x_	37.7%
		456.9 (462.4)	Ti^2+^	25.7%
		458.3 (463.9)	Ti^3+^	13.6%
		459.7 (465.5)	Ti-O	23.0%
	TiO_2_/Ti_3_C_2_T_x−_30 wt.%	455.2 (460.8)	C-Ti-T_x_	35.3%
		456.4 (462.3)	Ti^2+^	19.8%
		457.8 (464.3)	Ti^3+^	10.4%
		459.5 (465.8)	Ti-O	34.5%
O 1s	Ti_3_C_2_T_x_	530.7	O-Ti	21.6%
		533.3	-OH	72.4%
		534.8	O-C/O=C	6.0%
	TiO_2_/Ti_3_C_2_T_x−_30 wt.%	530.7	O-Ti	51.7%
		532.5	-OH	37.8%
		534.3	O-C/O=C	10.5%

## Data Availability

Data are contained within the article and Supplementary Materials.

## Supplementary Materials

The following supporting information can be downloaded via this link: https://www.mdpi.com/article/10.3390/ma17010072/s1, Figure S1: TEM image of TiO_2_ hollow sphere. Figure S2: AFM characterization of Ti_3_C_2_T_x_ MXene flakes. Figure S3: Prepared flexible TiO_2_-MXene film. Figure S4: Stress–strain curves of Ti_3_C_2_T_x_ and 30 wt.% TiO_2_-Ti_3_C_2_T_x_ composites. Figure S5: XRD patterns of composite materials with different TiO_2_ contents. Figure S6: XRD patterns of composite materials with different TiO_2_ contents. Figure S7: Performance of 30 wt.% TiO_2_-Ti_3_C_2_T_x_ with other Ti_3_C_2_T_x_ EMI shielding materials, where the red dot represents the samples of TiO_2_-Ti_3_C_2_T_x_. Table S1: Relative contents of the corresponding functional group/valence bonds of each element in MXene composite TiO_2_ material. Table S2: EMI shielding properties comparison of 30 wt.% TiO_2_-Ti_3_C_2_T_x_ with other shielding materials.

## Appendix A

### Appendix A.1. Materials

Ti_3_AlC_2_ MAX phase powder, with a particle size < 400 μm, was purchased from Xinxi Technology Co., Ltd. (Foshan, China). Lithium fluoride (LiF, 98.5%) was purchased from Alfa aesar. Hydrochloric acid (HCl, 37%) was obtained from Fisher Scientific. Anhydrous ethanol, TiO_2_, and ferric oxide were obtained from the Korea Darong chemical company. Distilled water with a resistivity of 10^6^ Ω/cm was used throughout the experiment. The product passed through a polypropylene membrane (pore size 0.064 μm) after vacuum-assisted filtration to obtain layered samples. The purchased reagents were of analytical grade and could be used without further purification.

### Appendix A.2. Synthesis of Ti_3_C_2_T_x_

Ti_3_C_2_T_x_ MXene was synthesized by selectively etching the aluminum elements of the layered precursor Ti_3_AlC_2_ MAX phase. In the experiment, a mixture of hydrochloric acid (HCl) and lithium fluoride (LiF) was used to etch the MAX phase of the ternary compound precursor. Lithium fluoride (2 g) was added to a 100 mL polyethylene beaker, and 20 mL hydrochloric acid was added to dissolve it. After the sample wholly dissolved, 1 g Ti_3_AlC_2_ powder was gradually added to the solution to avoid heat accumulation. Subsequently, the mixture was continuously stirred at 35 °C for 24 h. The product was washed (5–6 times) via centrifugation (3500 rpm, 5 min) with deionized water (DI) until a stable suspension (pH~6) of a dispersed single layer or a few layers of Ti_3_C_2_T_x_ flakes was obtained. The obtained dispersion was stored in an inert environment to avoid the oxidation of Ti_3_C_2_T_x_ sheets for subsequent use.

### Appendix A.3. Synthesis of TiO_2_ and Hollow Spheres

We added 3.3 mL of water, 23 mL of ethanol, and 0.62 mL of ammonia into a 40 mL beaker, stirred evenly, slowly added 1.06 mL of tetraethyl silicate, stirred at room temperature for 8 h, and then centrifuged to obtain a white precipitate. We washed it with ethanol several times and dried it in an oven at 60 °C to obtain SiO_2_ balls with good dispersion and uniform size. We then took 0.2 g of the above-prepared SiO_2_ ball and dispersed it in 150 mL of ethanol to form a uniform suspension. We added 0.9 mL of ammonia with a mass fraction of 25% to the suspension and evenly stirred it. We slowly added 2 mL of tertbutyl titanite (TBT) within 10 min, reacted it at 45 °C for 24 h, and then centrifuged it to obtain a white precipitate. We then washed it with ethanol several times and dried it in an oven at 60 °C, thus identifying SiO_2_@TiO_2_ precursor core–shell nanocomposites. The prepared SiO_2_@TiO_2_ core–shell nanocomposites were ultrasonically dispersed in 20 mL of ultrapure water. After we formed a uniform suspension, 1 mL of 2.5 m sodium hydroxide solution was added, stirred at room temperature for 8 h, centrifuged to obtain white precipitates, and then cleaned several times with ultrapure water and ethanol. The precipitate was dried in an oven at 60 °C to obtain TiO_2_ Nano hollow spheres.

### Appendix A.4. Fabrication of Ti_3_C_2_T_x_/SiO_2_ Nanocomposite Films

We took a certain amount of prepared TiO_2_ nano hollow spheres and dispersed them in 20 mL of ultrapure water via ultrasound. We added the obtained suspension into Ti_3_C_2_T_x_ dispersion and stirred evenly. Then, we slowly poured the mixed suspension into the vacuum suction bottle for suction filtration. After suction filtration, we transferred the obtained film to a vacuum-drying oven and dried it at room temperature for 12 h to obtain Ti_3_C_2_T_x_/TiO_2_ composite film. The TiO_2_ hollow sphere was attached to the middle of the MXene layer through mechanical meshing. The product was freeze-dried in an inert atmosphere to obtain the final product.

### Appendix A.5. Characterizations

The micro-morphology, hollow sphere size distribution, and product morphology of the Ti_3_C_2_T_x_/TiO_2_ composites were analyzed using a field emission scanning electron microscope (SEM) and transmission electron microscopy (TEM; F20 G2, FEI). The X-ray diffraction pattern adopts a 40 kV–44 MV cu-k α radiation source, obtained using a step scan of 0.02°. X-ray photoelectron spectroscopy (XPS) was obtained using a versa probe spectrometer (phi 5000, ULVAC phi). EMI shielding measurements were performed in the X-band (8.2–12.4 GHz) frequency range using a vector network analyzer (Agilent Technologies ena5071c).
