# Supplementary material for: Self-Assembly TiO2-Ti3C2Tx Ball–Plate Structure for Highly Efficient Electromagnetic Interference Shielding
Source: Materials (Basel). 2023 Dec 22;17(1):72. doi: 10.3390/ma17010072 (PMC10779825; doi:10.3390/ma17010072)
Supplement: Supplementary file 1 [file materials-17-00072-s001.zip › materials-2778791-supplementary.pdf]

Self-Assembly TiO_2-$Ti_3C_2T_x$ Ball–Plate Structure for Highly Efficient Electromagnetic Interference Shielding

Zhen Zhang [1], Xingyang Ning [1], Bin Liu [2], Jian Zhou [1] and Zhimei Sun [1,*]

1 School of Materials Science and Engineering, Beihang University, Beijing 100191, China; by1901053@buaa.edu.cn (Z.Z.); 13032495593@163.com (X.N.); jzhou@buaa.edu.cn (J.Z.)
2 School of Integrated Circuit Science and Engineering, Beihang University, Beijing 100191, China; binliu@buaa.edu.cn
* Correspondence: zmsun@buaa.edu.cn; Tel.: +86-188-1092-2621

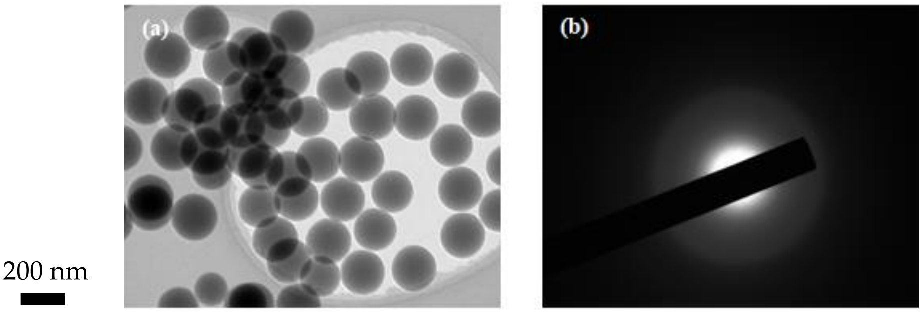

(a) **(b)**

Figure S1. TEM image of TiO_2 hollow sphere

The TEM characterization shows that titanium dioxide hollow spheres have typical amorphous properties and are uniform in size

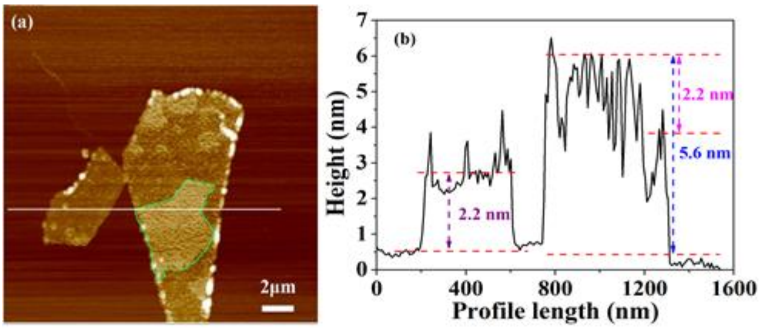

(a) **(b)**

Figure S2. AFM characterization of MXene flakes

AFM characterization shows that the obtained MXene sheets have thicknesses of 2.2 nm and 5.6 nm, which are consistent with the calculated thicknesses of the monolayer and bilayer $Ti_3C_2T_x$ materials.

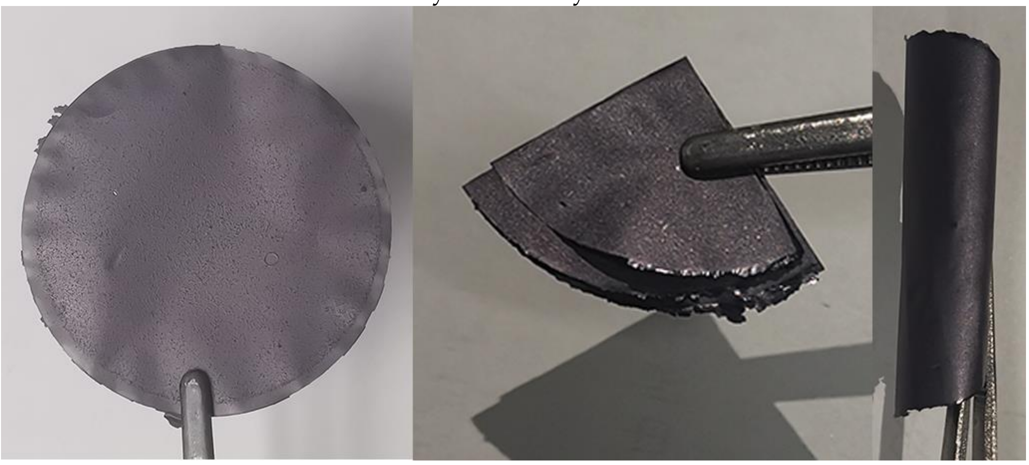

Figure S3. Prepared flexible TiO_2-MXene film

The circular thin layer of shielding material obtained after vacuum filtration.

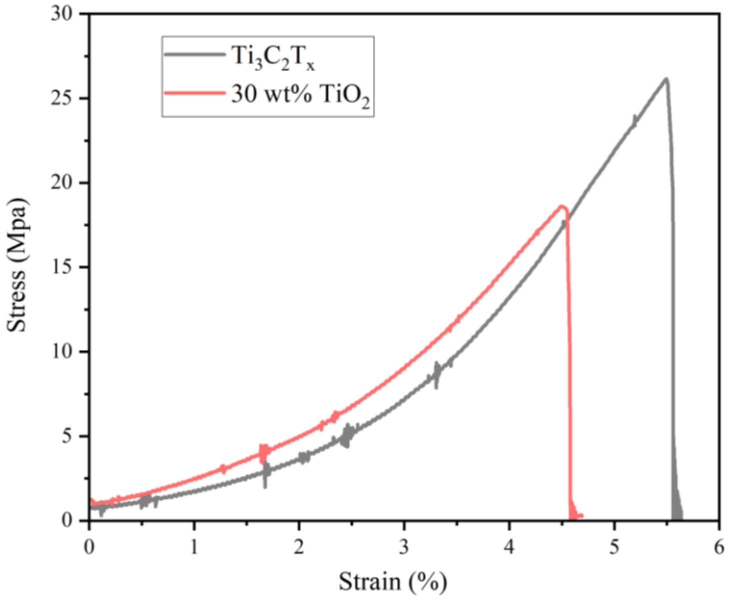

Figure S4. Stress-strain curves of $Ti_3C_2T_x$ and 30 wt.% TiO_2- $Ti_3C_2T_x$ composites

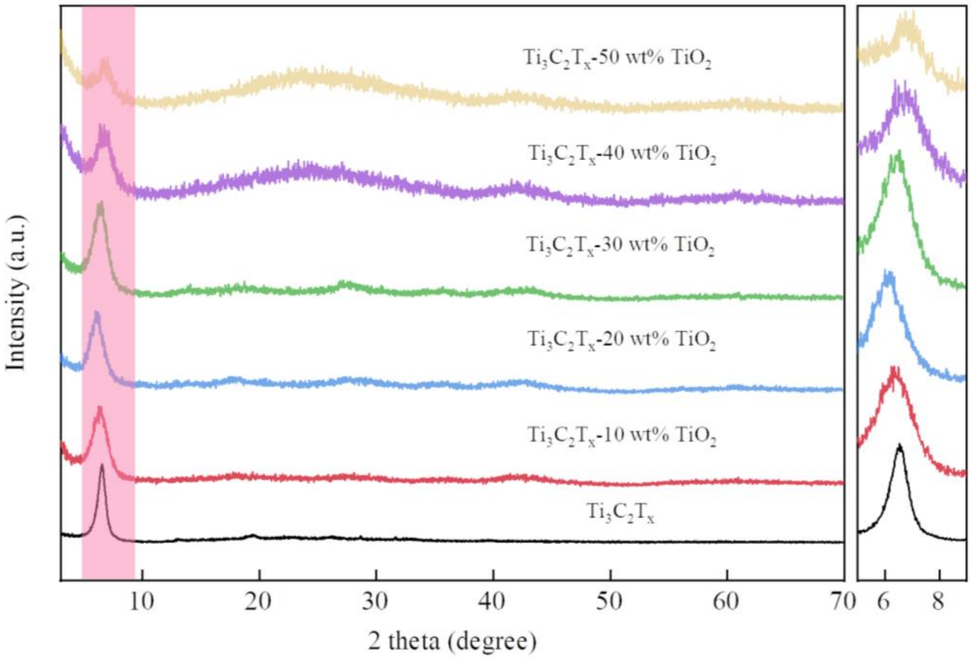

Figure S5. XRD patterns of composite materials with different TiO_2 contents

The XRD patterns of the composites with different titanium dioxide contents clearly show the characteristic peak patterns of the matrix and the filler.

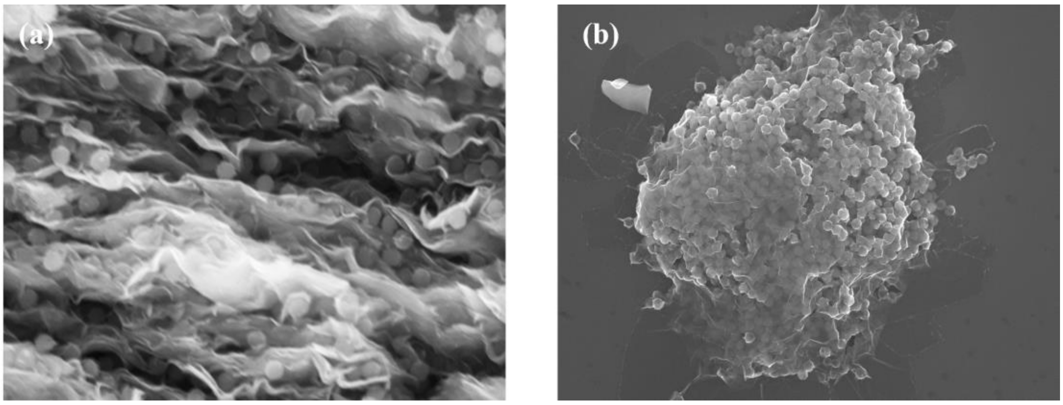

(a) **(b)**

Figure S6. SEM images of 50wt.% TiO_2-MXene composites.

Scanning electron micrograph of 50wt.% TiO_2-MXene, the agglomeration of interlayer and surface titanium dioxide hollow spheres can be clearly seen.

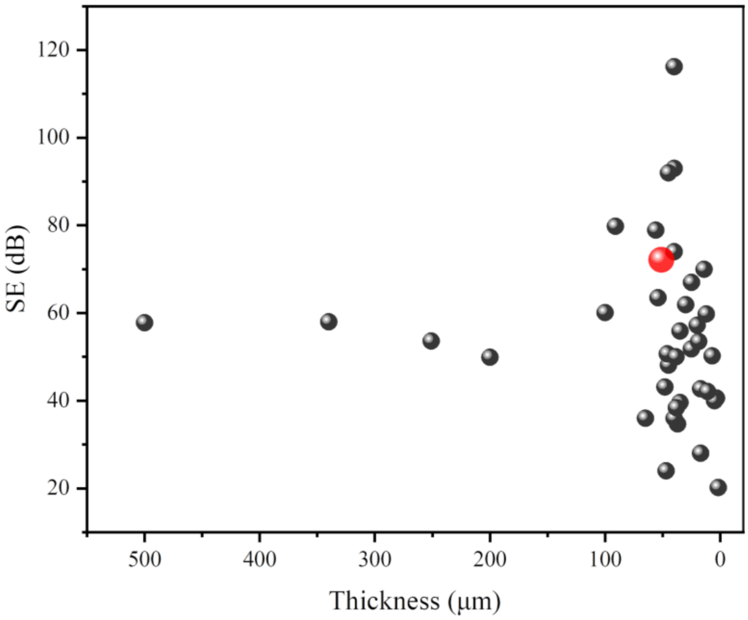

Figure S7. Performance of 30 wt.% TiO_2- $Ti_3C_2T_x$ with other $Ti_3C_2T_x$ EMI shielding materials, the red dot represents the samples of TiO_2- $Ti_3C_2T_x$.

Table.S1 Relative content of corresponding functional group/valence bond of each element in MXene composite TiO_2 material

Sample(element)	Binding energy (eV)	Functional group/ valence bond	Relative content
$Ti_3C_2T_x$ Ti 2p	455.5 (461.2)	C-Ti-Tx	37.7%
	456.9 (462.4)	Ti^{2+}	25.7%
	458.3 (463.9)	Ti^{3+}	13.6%
	459.7 (465.5)	Ti-O	23.0%
TiO_2/$Ti_3C_2T_x$-30wt.% 2p	455.2 (460.8)	C-Ti-Tx	35.3%
	456.4 (462.3)	Ti^{2+}	19.8%
	457.8 (464.3)	Ti^{3+}	10.4%
	459.5 (465.8)	Ti-O	34.5%

$Ti_3C_2T_x$ C 1s	282.0	C-Ti-T_x	5.1%
	284.8	C-C	91.1%
	286.7	C-O/C=O/C-F	3.8%
$TiO_2/Ti_3C_2T_x$-30wt.% C 1s	281.9	C-Ti-T_x	5.6%
	284.8	C-C	83.3%
	286.3	C-O/C=O/C-F	11.1%
$Ti_3C_2T_x$ O 1s	530.7	O-Ti	21.6%
	533.3	-OH	72.4%
	534.8	O-C/O=C	6.0%
$TiO_2/Ti_3C_2T_x$-30wt.% O 1s	530.7	O-Ti	51.7%
	532.5	-OH	37.8%
	534.3	O-C/O=C	10.5%

Table S2 EMI shielding properties comparison of 30 wt.% TiO_2- $Ti_3C_2T_x$ with other shielding materials

Composition	Structure design	Thickness [μm]	EMI SE [dB]	SSE/*t* [dB cm² g⁻¹]
**30 wt.% TiO_2-$Ti_3C_2T_x$ composites**	**Ball-plated**	**50**	**72**	**8372 (~1.72g cm⁻³)**
Heat-treated Ti_3CNT_x	Porous	40	116.2	—
Heat-treated $Ti_3C_2T_x$		40	93.0	—
$Ti_3C_2T_x$ /NR		251	53.6	—
$Ti_3C_2T_x$	Layered	45	92	—
$Ti_3C_2T_x$ /SWCNT		56	78.9	15 236.1
$Ti_3C_2T_x$		14	70	—
$Ti_3C_2T_x$ /NR		54	63.5	—
$Ti_3C_2T_x$ /cellulose		12.1	59.8	18 637.1
$Ti_3C_2T_x$ /CNF/MWCNT		25.1	51.8	12 011.8
$Ti_3C_2T_x$ /C60		18.7	53.5	14 953.2
$Ti_3C_2T_x$ /GO		7	50.2	—
$Ti_3C_2T_x$ /ANF		3.2	40.6	50 491.0
V_2CT_x		5	≈40	—
$Ti_3C_2T_x$ /Chitosan		37	34.7	—
$Ti_3C_2T_x$ -AgNW/ANF	Bilayered	91	79.8	5 379.9
$Ti_3C_2T_x$ /CNF		100	60.1	—

$Ti_3C_2T_x$ -AgNW/ANF		45	48.1	8 907.4
$Ti_3C_2T_x$ /MMT	"Brick-and-	25	67	10 156.3
$Ti_3C_2T_x$ /ANF	mortar"	20	57.2	36 641.9
$Ti_3C_2T_x$ /cellulose/AgNP		46	50.7	—
$Ti_3C_2T_x$ /PVA		48	43.1	—
$Ti_3C_2T_x$ /AgNW		17	42.7	16 724
$Ti_3C_2T_x$ /PEDOT:PSS		11.1	42.1	—
$Ti_3C_2T_x$ /nanocellulose		65	36	—
$Ti_3C_2T_x$ /PANI		40	36	—
$Ti_3C_2T_x$ /ANF		17	28	—
$Ti_3C_2T_x$ /CNF		47	24	2 647.0
$Ti_3C_2T_x$ /cellulose/AgNW	Sandwich	30	61.9	—
$Ti_3C_2T_x$ /cellulose/AgNW		35	55.9	10 647.6
$Ti_3C_2T_x$ /MMT		38.4	50	—
$Ti_3C_2T_x$ /NR/CNT		200	49.9	1 885.5
$Ti_3C_2T_x$ /CNF		35	39.6	7 029.0
$Ti_3C_2T_x$ /cellulose/CNT		38	38.4	8 020.0
$Ti_3C_2T_x$ /PS	Segregated	≈40	74	—
$Ti_3C_2T_x$ /CNF/FeCo	Gradient	340	58	—
$Ti_3C_2T_x$ /woodpulp/MTMS	Decorated fabric	≈500	57.8	—
$Ti_3C_2T_x$ /cellulose	Janus	1.73	20.2	69 455.2

EXPERIMENT DETAILS

1. Materials

Ti_3AlC_2 MAX phase powder, particle size < 400 μm, purchased from Xinxi Technology Co., Ltd. (Foshan, China). Lithium fluoride (LiF, 98.5%) was purchased from Alfa aesar. Hydrochloric acid (HCl, 37%) was obtained from Fisher Scientific. Anhydrous ethanol, TiO_2, and ferric oxide were obtained from the Korea Darong chemical company. Distilled water with a resistivity of 10^6 Ω /cm was used throughout the experiment. The product passes through a polypropylene membrane (pore size 0.064 μm) after vacuum-assisted filtration to obtain layered samples. The purchased reagents are of analytical grade and can be used without further purification.

2. Synthesis of $Ti_3C_2T_x$

$Ti_3C_2T_x$ MXene was synthesized by selectively etching the aluminum elements of the layered precursor Ti_3AlC_2 MAX phase. In the experiment, a mixture of hydrochloric acid (HCl) and lithium fluoride (LiF) was used to etch the MAX phase of the ternary compound precursor. Lithium fluoride (2 g) was added to a 100 ml polyethylene beaker and 20 ml hydrochloric acid was added to dissolve it. After completely dissolved, 1g Ti_3AlC_2 powder was gradually added to the solution to avoid heat accumulation. Subsequently, the mixture was continuously stirred at 35 ° C for 24 hours. The product was washed (5-6 times) by centrifugation (3500 rpm, 5 min) with deionized water (DI) until a stable suspension (pH~6) of dispersed single-layer or few layers of $Ti_3C_2T_x$ flakes was obtained. The obtained dispersion was stored in an inert environment to avoid oxidation of $Ti_3C_2T_x$ sheets for subsequent use.

3． Synthesis of TiO_2 and hollow spheres

Added 3.3 ml of water, 23 ml of ethanol, and 0.62 ml of ammonia into a 40 ml beaker, stir evenly, then slowly added 1.06 ml of tetraethyl silicate, stirred at room temperature for 8 h, and then centrifuged to obtain a white precipitate. Washed it with ethanol several times and dried it in an oven at 60 ℃ to obtain SiO_2 balls with good dispersion and uniform size. Taken 0.2 g of the above-prepared SiO_2 ball and dispersed in 150 ml of ethanol to form a uniform suspension. Added 0.9 ml of ammonia with a mass fraction of 25% to the suspension and stirred it evenly. Slowly added 2 ml of tertbutyl titanite (TBT) within 10 minutes, reacted at 45 ℃ for 24 hours, and then centrifuged to obtain a white precipitate. Washed it with ethanol several times and dried it in an oven at 60 ℃, thus getting SiO_2@TiO_2 precursor core-shell nanocomposites. The prepared SiO_2@TiO_2 core-shell nanocomposites were ultrasonically dispersed in 20 ml of ultrapure water. After formed a uniform suspension, 1 ml of 2.5m sodium hydroxide solution was added, stirred at room temperature for 8 h, centrifuged to obtain white precipitates, and then cleaned several times with ultrapure water and ethanol. The precipitate was dried in an oven at 60 ℃ to obtain TiO_2 Nano hollow spheres.

4. Fabrication of $Ti_3C_2T_x/SiO_2$ nanocomposite films

Taken a certain amount of prepared TiO_2 nano hollow spheres and dispersed them in 20 ml ultrapure water by ultrasound. Added the obtained suspension into $Ti_3C_2T_x$ dispersion and stirred evenly. Then slowly poured the mixed suspension into the vacuum suction bottle for suction filtration. After suction filtration, transferred the obtained film to a vacuum drying oven and dried at room temperature for 12 hours to obtain $Ti_3C_2T_x$ / TiO_2 composite film. The TiO_2 hollow sphere was attached to the middle of the MXene layer through mechanical meshing. The product was freeze-dried in an inert atmosphere to obtain the final product.

5．Characterizations

The micro-morphology, hollow sphere size distribution, and product morphology of $Ti_3C_2T_x/TiO_2$ composites were analyzed by field emission scanning electron microscope (SEM) and transmission electron microscopy (TEM; F20 G2, FEI). The X-ray diffraction pattern adopts a 40 kV - 44 MV cu-k α radiation source, obtained in a step scan of 0.02°. X-ray photoelectron spectroscopy (XPS) was obtained by a versa probe spectrometer (phi 5000, ULVAC phi). EMI shielding measurements were performed in the X-band (8.2 – 12.4 GHz) frequency range using a dual port wr-90 rectangular waveguide network analyzer (Agilent Technologies ena5071c).
